# Supplementary material for: Macronutrient Intake in Adults Diagnosed with Metabolic Syndrome: Using the Health Examinee (HEXA) Cohort
Source: Nutrients. 2021 Dec 14;13(12):4457. doi: 10.3390/nu13124457 (PMC8706324; doi:10.3390/nu13124457)
Supplement: Supplementary file 1 [file nutrients-13-04457-s001.zip › nutrients-1467631-supplementary.pdf]

Table S1. Distribution of demographic, lifestyle and clinical characteristics of participants according to quartiles of energy intake.

	Men (n = 43 850)					Women (n = 86 573)				
	**Q 1**	**Q 2**	**Q 3**	**Q4**	***P*-value**	**Q 1**	**Q 2**	**Q3**	**Q 4**	***P*-value**
Total energy intake (kcal/day, median)	1351	1654	1931	2416	<0.001	1144	1509	1786	2237	<0.001
Age (years, median)	55.0	54.0	53.0	51.0	<0.001	53.0	53.0	52.0	50.0	<0.001
Body mass index (kg/m^2, median)	24.1	24.2	24.4	24.6	<0.001	23.2	23.3	23.4	23.4	<0.001
Marital status (yes, %)	92.8	94.4	95.3	94.4	<0.001	83.2	85.9	88.3	89.8	<0.001
Education (≥12 years, %)	41.3	42.1	44.9	47.2	<0.001	21.2	21.4	24.6	29.5	<0.001
Family income (≥$3,000/month, %)	46.3	47.5	51.6	53.3	<0.001	40.8	41.1	44.9	49.1	<0.001
Occupied (yes, %)	77.1	79.9	82.7	85.4	<0.001	37.3	39.3	40.2	41.7	<0.001
Current smoker (yes, %)	30.6	30.5	31.1	35.3	<0.001	3.01	2.20	2.15	1.98	<0.001
Current drinker (yes, %)	69.8	72.7	74.1	74.8	<0.001	30.0	28.7	30.8	33.0	<0.001
Regular exercise (yes, %)	53.6	56.9	58.7	59.3	<0.001	49.2	48.7	52.3	54.5	<0.001
Hypertension (yes, %)	53.2	52.7	52.3	51.0	<0.001	38.1	39.2	36.7	33.6	<0.001
Diabetes (yes, %)	35.5	35.2	33.9	32.9	<0.001	20.5	20.2	19.2	17.6	<0.001
Dyslipidemia (yes, %)	40.45	40.00	40.11	40.05	0.610	28.41	28.28	27.30	25.80	<0.001
Macronutrient intake (% of energy)										
Carbohydrate	74.4	74.1	71.4	68.8	<0.001	72.8	73.6	71.8	69.2	<0.001
Protein	12.3	12.4	13.2	13.9	<0.001	13.4	12.9	13.5	14.3	<0.001
Fat	11.6	11.7	14.0	16.4	<0.001	12.9	12.2	13.8	16.1	<0.001

Comparisons were made by Mantel-Haenszel chi-square test for categorical variables, and general linear regression for continuous variables.

Table S2. Distribution of MetS cases by sex and age group.

	Male		Female	
**Age group**	**Cases**	**Controls**	**Cases**	**Controls**
40-49	3769	11268	29387	3962
50-59	4861	11490	26061	9357
60-69	4010	8452	10097	7709

Table S3. Median macronutrient intake according to MetS and its components stratified by age group.

	MetS case/control[a]	Abdominal obesity case/control[a]	High triglyceride case/control[a]	Low HDL-c case/control[a]	High blood pressure case/control[a]	Hyperglycemia case/control[a]
**Men**						
Energy (kcal/day)						
40-49	1875 (1590,2241)/ 1846 (1568,2217)	1904 (1614,2285)/ 1836 (1559,2203)	1860 (1576,2226)/ 1851 (1571,2225)	1854 (1568,2215)/ 1855 (1574,2230)	1865 (1570,2234)/ 1844 (1574,2217)	1851 (1566,2225)/ 1856 (1575,2226)
50-59	1778 (1525,2111)/ 1774 (1511,2096)	1809 (1541,2152)/ 1763 (1505,2081)	1778 (1522,2112)/ 1774 (1511,2089)	1772 (1518,2106)/ 1777 (1515,2098)	1770 (1516,2091)/ 1783 (1515,2110)	1769 (1514,2093)/ 1780 (1517,2105)
60-69	1727 (1473,2032)/ 1711 (1471,2013)	1751 (1500,2069)/ 1700 (1459,1994)	1720 (1473,2017)/ 1714 (1470,2019)	1709 (1463,1997)/ 1719 (1474,2025)	1716 (1473,2021)/ 1716 (1470,2014)	1710 (1467,2011)/ 1721 (1474,2024)
Carbohydrate (g/day)						
40-49	327 (280,386)/ 324 (279,382)	330 (282,394)/ 322 (278,379)	324 (279,383)/ 324 (280,383)	327 (279,383)/ 324 (279,383)	325 (279,383)/ 324 (279,383)	322 (277,381)/ 325 (280,383)
50-59	316 (277,368)/ 317 (276,366)	321 (278,375)/ 315 (275,364)	317 (276,370)/ 317 (276,365)	318 (279,369) /316 (275,366)	315 (276,364)/ 318 (276,370)	314 (276,364)/ 318 (276,369)
60-69	312 (274,359)/ 311 (273,356)	315 (276,364)/ 310 (272,354)	312 (275,358)/ 312 (273,356)	313 (275,358)/ 311 (273,356)	311 (273,357)/ 312 (273,356)	309 (272,355)/ 313 (274,359)
Protein (g/day)						
40-49	63.1 (51,79)/ 61.4 (49,78)	64.3 (52,81)/ 60.7 (49,77)	62.0 (49,78)/ 61.6 (49,78)	61.2 (49,77)/ 61.9 (49,78)	62.5 (50,78)/ 61.3 (49,78)	62.5 (50,78)/ 61.5 (49,78)
50-59	59.3 (47,74)/ 58.3 (46,73)	60.3 (48,76)/ 57.8 (46,72)	58.9 (47,73)/ 58.3 (46,73)	57.6 (46,72)/ 58.9 (47,74)	58.8 (47,73)/ 58.4 (47,73)	59.0 (47,73)/ 58.4 (47,73)
60-69	56.4 (45,71)/ 55.1 (44,70)	57.5 (46,73)/ 54.6 (44,69)	55.9 (44,70)/ 55.3 (44,70)	54.8 (44,69)/ 55.7 (45,70)	55.7 (44,70)/ 55.2 (45,70)	55.9 (45,70)/ 55.3 (44,70)
Fat (g/day)						
40-49	31.5 (23,43)/ 30.7 (22,42)	32.5 (23,45)/ 30.3 (22,42)	31.1 (22,43)/ 30.8 (22,42)	30.4 (22,41)/ 31.1 (22,43)	30.9 (22,42)/ 30.9 (22,43)	31.0 (22,43)/ 30.9 (22,42)
50-59	26.8 (19,37)/ 26.8 (19,37)	27.7 (20,39)/ 26.4 (18,37)	26.8 (19,37)/ 26.8 (19,37)	25.6 (18,36)/ 27.1 (19,38)	26.6 (19,37)/ 26.9 (19,37)	26.6 (19,37)/ 26.9 (19,37)
60-69	24.4 (17,35)/ 23.9 (17,34)	25.5 (18,36)/ 23.4 (16,33)	24.3 (17,34)/ 23.9 (17,34)	23.0 (16,33)/ 24.4 (17,34)	24.0 (16,34)/ 24.2 (17,34)	24.2 (17,34)/ 24.0 (16,34)
**Women**						
Energy (kcal/day)						

40-49	1699 (1404,2044)/ 1703 (1393,2039)	1722 (1418,2062)/ 1693 (1386,2030)	1700 (1393,2051)/ 1703 (1395,2038)	1709 (1407,2043)/ 1699 (1388,2039)	1685 (1392,2021)/ 1707 (1396,2044)	1691 (1395,2023)/ 1704 (1395,2042)
50-59	1632 (1367,1937)/ 1635 (1342,1947)	1649 (1377,1958)/ 1621 (1329,1935)	1629 (1352,1940)/ 1635 (1350,1946)	1623 (1357,1929)/ 1640 (1346,1954)	1630 (1356,1937)/ 1636 (1346,1949)	1627 (1347,1939)/ 1636 (1351,1945)
60-69	1564 (1310,1842)/ 1571 (1308,1865)	1573 (1318,1861)/ 1560 (1292,1850)	1567 (1307,1854)/ 1569 (1308,1857)	1553 (1298,1834)/ 1581 (1316,1869)	1568 (1311,1848)/ 1568 (1303,1863)	1557 (1288,1830)/ 1573 (1316,1865)
Carbohydrate (g/day)						
40-49	307 (252,359)/ 305 (246,358)	309 (252,361)/ 303 (244,357)	307 (248,362)/ 305 (246,357)	308 (253,361)/ 303 (244,357)	305 (248,357)/ 305 (246,358)	303 (247,355)/ 305 (246,358)
50-59	302 (253,349)/ 299 (243,349)	303 (253,350)/ 297 (240,347)	301 (249,350)/ 299 (244,348)	300 (250,347)/ 299 (243,349)	300 (248,349)/ 299 (244,349)	300 (246,347)/ 300 (245,349)
60-69	295 (247,339)/ 293 (242,338)	295 (248,340)/ 292 (239,337)	295 (247,341)/ 293 (243,337)	293 (245,337)/ 294 (244,340)	294 (246,339)/ 293 (243,338)	292 (241,335)/ 295 (246,340)
Protein (g/day)						
40-49	55.7 (44,71)/ 56.1 (44,71)	57.2 (45,72)/ 55.7 (44,70)	55.6 (44,71)/ 56.2 (44,71)	55.9 (44,71)/ 56.2 (44,71)	55.3 (44,70)/ 56.3 (44,71)	56.1 (44,71)/ 56.1 (44,71)
50-59	52.4 (42,67)/ 53.3 (42,68)	53.5 (42,68)/ 52.7 (41,67)	52.2 (41,66)/ 53.3 (42,68)	52.1 (41,66)/ 53.5 (42,68)	52.7 (42,67)/ 53.3 (42,68)	52.6 (41,67)/ 53.1 (42,67)
60-69	49.3 (39,62)/ 50.0 (40,64)	49.7 (39,63)/ 49.6 (39,63)	49.4 (39,62)/ 49.8 (39,63)	48.9 (39,61)/ 50.5 (40,64)	49.6 (39,63)/ 49.7 (39,63)	49.2 (39,62)/ 49.9 (39,63)
Fat (g/day)						
40-49	25.3 (18,36)/ 26.5 (19,37)	26.7 (19,37)/ 26.3 (19,37)	25.3 (17,36)/ 26.5 (19,37)	25.5 (18,36)/ 26.8 (19,37)	25.2 (18,36)/ 26.7 (19,37)	26.2 (18,37)/ 26.4 (19,37)
50-59	21.2 (15,30)/ 22.8 (16,32)	22.3 (15,32)/ 22.4 (16,32)	21.4 (15,31)/ 22.8 (16,32)	21.2 (14,30)/ 23.1 (16,33)	21.8 (15,31)/ 22.8 (16,32)	21.8 (15,31)/ 22.5 (16,32)
60-69	18.4 (12,27)/ 19.6 (14,29)	18.9 (13,27)/ 19.4 (13,28)	18.7 (12,27)/ 19.3 (13,28)	18.0 (12,26)/ 19.9 (14,29)	19.0 (13,27)/ 19.3 (13,28)	18.7 (13,27)/ 19.3 (13,28)

HDL-c: High density lipoprotein cholesterol. [a] Median (interquartile range, Q1,Q3).

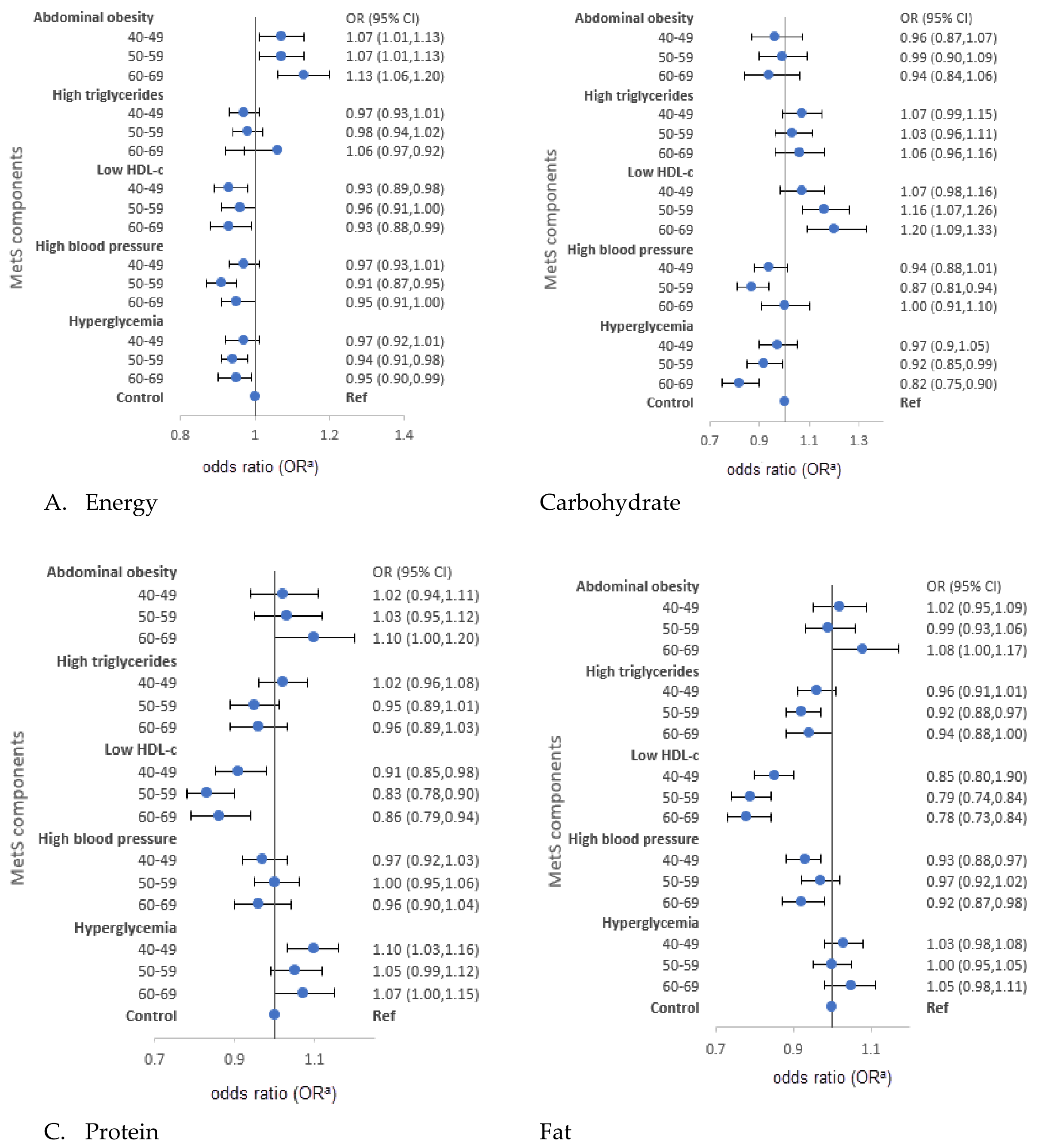

Figure S1. Odds ratios and 95% CI of MetS components stratified by age-group in men. (A) energy intake, (B) carbohydrate intake, (C) protein intake, (D) fat intake. HDL-c: High density lipoprotein cholesterol. [a] Adjusted for for age, body mass index, marital status, education, family income, occupation, smoking, drinking, regular exercise and energy intake.

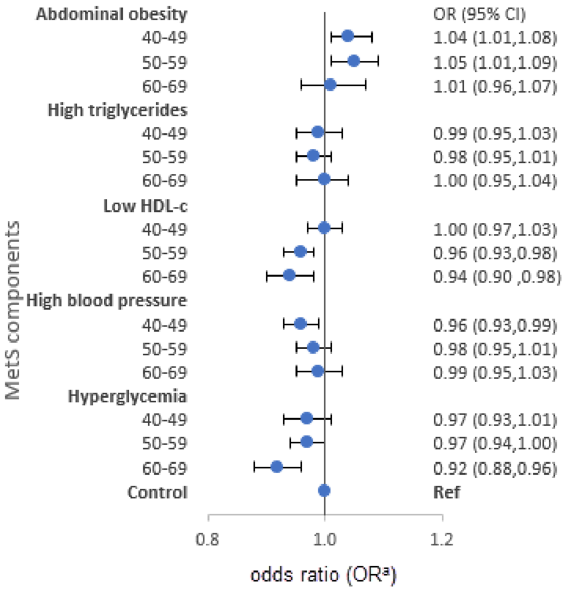

A. Energy

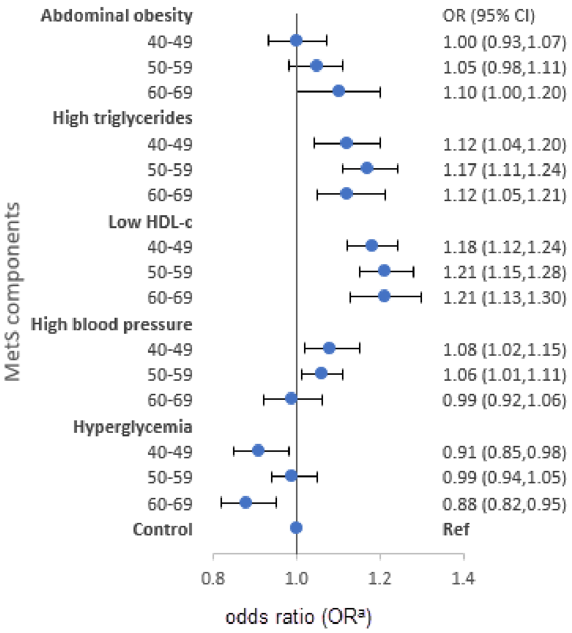

Carbohydrate

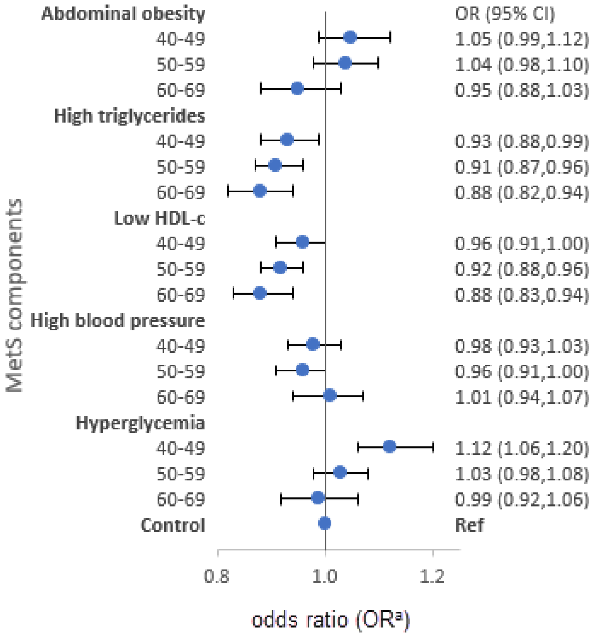

C. Protein

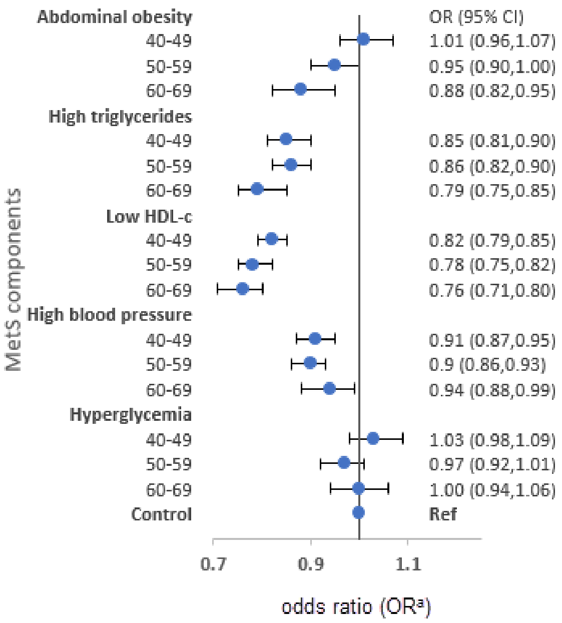

Fat

Figure S2. Odds ratios and 95% CI of MetS components stratified by age-group in women. (A) energy intake, (B) carbohydrate intake, (C) protein intake, (D) fat intake. HDL-c: High density lipoprotein cholesterol. [a] Adjusted for for age, body mass index, marital status, education, family income, occupation, smoking, drinking, regular exercise, energy intake and menopausal status.

Table S4. Median macronutrient intake according to MetS and its components stratified by macronutrient source.

	MetS	Abdominal obesity	High triglyceride	Low HDL-c	High blood pressure	Hyperglycemia
	**Case/control[a]**	**Case/control[a]**	**Case/control[a]**	**Case/control[a]**	**Case/control[a]**	**Case/control[a]**
**Men**						
Carbohydrate (g/day)						
Plant	309 (270,358)/ 308 (269,356)	312 (272,364)/ 306 (268,353)	309 (270,359)/ 307 (269,355)	309 (271,358)/ 307 (269,356)	307 (269,354)/ 309 (269,358)	306 (268,353)/ 309 (270,358)
Animal	6.2 (2,14)/ 7.2 (3,15)	6.7 (2,15)/ 7.0 (2,15)	6.4 (2,14)/ 7.2 (3,15)	6.5 (2,14)/ 7.0 (2,15)	6.5 (2,14)/ 7.4 (3,15)	6.3 (2,14)/ 7.2 (3,15)
Protein (g/day)						
Plant	36.9 (31,44)/ 36.5 (31,44)	37.4 (31,45)/ 36.3 (31,43)	36.9 (31,44)/ 36.4 (31,43)	36.5 (31,44)/ 36.6 (31,44)	36.7 (31,44)/ 36.5 (31,44)	36.5 (31,44)/ 36.6 (31,44)
Animal	21.3 (14,32)/ 21.0 (13,32)	22.3 (14,33)/ 20.6 (13,31)	21.2 (14,32)/ 20.9 (13,32)	20.1 (13,31)/ 21.3 (14,32)	20.9 (13,31)/ 21.2 (14,32)	21.1 (13,32)/ 21.0 (13,32)
Fat (g/day)						
Plant	10.8 (8,15)/ 10.9 (8,15)	11.1 (8,16)/ 10.7 (8,15)	11.0 (8,15)/ 10.7 (8,15)	10.6 (8,15)/ 10.9 (8,15)	10.7 (8,15)/ 11.0 (8,15)	10.4 (7,15)/ 11.1 (8,15)
Animal	15.4 (10,24)/ 15.5 (10,24)	16.2 (10,25)/ 15.2 (9,23)	15.5 (10,24)/ 15.4 (10,24)	14.4 (9,23)/ 15.7 (10,24)	15.2 (9,23)/ 15.7 (10,24)	15.3 (10,24)/ 15.5 (10,24)
**Women**						
Carbohydrate (g/day)						
Plant	290 (241,334)/ 290 (232,337)	292 (241,336)/ 288 (230,336)	290 (238,336)/ 290 (234,336)	291 (240,336)/ 289 (231,336)	289 (237,333)/ 290 (233,337)	288 (234,332)/ 290 (235,337)
Animal	7.8 (3,16)/ 9.8 (4,19)	8.7 (3,17)/ 9.8 (4,19)	8.6 (3,17)/ 9.7 (4,18)	8.2 (3,16)/ 9.9 (4,19)	8.8 (3,17)/ 9.8 (4,18)	8.6 (3,17)/ 9.7 (3,18)
Protein (g/day)						
Plant	33.7 (28,40)/ 33.8 (28,41)	34.0 (28,41)/ 33.7 (27,41)	33.7 (28,40)/ 33.8 (28,41)	33.7 (28,40)/ 33.8 (28,41)	33.6 (28,40)/ 33.9 (28,41)	33.5 (27,40)/ 33.8 (28,41)
Animal	17.5 (11,27)/ 19.6 (12,29)	18.7 (12,28)/ 19.4 (12,29)	17.8 (11,27)/ 19.5 (12,29)	18.0 (11,28)/ 19.7 (13,30)	18.0 (11,28)/ 19.7 (13,29)	18.3 (11,28)/ 19.3 (12,29)
Fat (g/day)						
Plant	8.3 (6,12)/	8.7 (6,13)/	8.6 (6,12)/	8.6 (6,12)/	8.6 (6,12)/	8.5 (6,12)/

	9.3 (7,13)	9.3 (6,13)	9.2 (6,13)	9.4 (7,13)	9.4 (7,14)	9.2 (6,13)
Animal	11.7 (7,19)/<br>13.7 (8,21)	12.7 (8,20)/<br>13.6 (8,21)	11.9 (7,19)/<br>13.6 (8,21)	12.2 (7,19)/<br>13.8 (9,21)	12.2 (7,19)/<br>13.8 (9,21)	12.4 (7,20)/<br>13.4 (8,21)

HDL-c: High density lipoprotein cholesterol. [a] Median (interquartile range, Q1,Q3).
